# Qualitative analysis of front-of package labeling policy interactions between stakeholders and Health Canada

**DOI:** 10.3389/fpubh.2023.982908

**Published:** 2023-04-06

**Authors:** Aalaa Jawad, Christine Mulligan, Natalie Savona, Mary R. L'Abbé

**Affiliations:** ^1^Faculty of Public Health and Policy, London School of Hygiene & Tropical Medicine, London, United Kingdom; ^2^Department of Nutritional Sciences, University of Toronto, Toronto, ON, Canada

**Keywords:** front-of-package labeling, nutrition labeling, food labeling, nutrition policy, consultation, obesity, industry, lobbying

## Abstract

**Background:**

Front-of-package labelling regulations proposed by Health Canada in their Healthy Eating Strategy (2016) were finally passed in 2022, but remain unimplemented. This study analyzed interactions that occurred between stakeholders and government related to this policy proposal to identify key themes and policy implications.

**Methods:**

A qualitative framework analysis was conducted on publicly available documents for stakeholder correspondences related to front-of-package that occurred between 2016 and 2019 in Health Canada’s Meetings and Correspondence on Healthy Eating database. Five sequential steps were applied: familiarization, identifying a thematic framework, indexing, charting, and mapping and interpretation. A complex systems (i.e., a dynamic system with multiple interconnecting components) lens was incorporated in the final step to deepen the analysis.

**Results:**

Hundred and seventy-three documents were included, the majority from industry stakeholders (*n* = 108, 62.4%). Three overarching themes were identified: industry trying to control the agenda and resist regulation; questioning the evidence supporting the policy and its impact on the agri-food industry; and dismissing the need and effectiveness of the policy. Incorporating a complex system lens found industry and non-industry stakeholders held markedly different perspectives on how cohesive the system defined by the front-of-package labelling policy was, and the policy impact on its stability. Economic and opportunity costs were the main trade-offs, and symbol misinterpretation considered an unintended consequence by industry. Finally, some stakeholders argued for wider policy scope incorporating more products, while others requested a narrower approach through exemptions.

**Conclusion:**

Interactions with industry stakeholders on health food policy proposals require careful consideration, given it may suit their interests to generate delays and policy discordance. Explicitly setting out the principles of engagement and actively encouraging non-industry stakeholder representation provides a more balanced approach to policy consultation and development.

## Introduction

Diet-related chronic non-communicable diseases continue to have a profound effect on health. Globally, dietary risks were responsible for 7.94 million deaths and 188 million disability-adjusted life-years among adults, as calculated by the Global Burden of Disease Study 2019 (1). In Canada 63.1% of adults (aged 18 or older) were classified as overweight or obese in 2018 (2) and high body mass index (BMI) was the most significant risk factor after tobacco for death and disability in Canada (3). Risk factors related to unhealthy diets and chronic disease are estimated to cost $26.7 billion in Canada annually (4).

Given the burden of disease generated by poor diets, a range of policy options have been deployed to mitigate them. The World Cancer Research Fund International NOURISHING database identifies a range of policies in place to promote healthy diets including nutritional labelling policies on foods such as front-of-package labelling (FOPL) (5, 6). It records this policy being implemented by over 50 governments globally including mandatory labelling in much of Latin America (6–8). FOPL provides visible nutritional information on the display surface of a product and can be presented as nutrient-specific systems focusing on a few key nutrients (such as fats, salt and sugar), or as summary systems that provide an overall nutritional score (9).

One aim of FOPL is to promote healthier choices by consumers. The literature on FOPL has assessed its role in outcomes such as healthier product identification, selection, purchasing, and consumption. One meta-analysis found that FOPL use resulted in easier identification of healthier foods and a smaller positive effect on consumer purchasing but limited evidence on consumption behaviours (10). Similarly, a second review found a significant overall effect of any FOPL compared to no-label for lower sugar and sodium content of purchases, but limited findings on consumption (11). Another aim of FOPL is to prompt the reformulation of products that would be required to display a FOPL to make their nutrition profile healthier, and there is evidence to show the impact of this both in Chile where a prospective study found a significant decrease in the proportion of products with any “high in” nutrient of concern from 51 to 44% (12), and in Australia and New Zealand where the Health Star Rating has prompted the reformulation of less healthy foods (i.e., with a lower number of stars) (13). Thus, the current evidence around FOPL leading manufacturers to reformulate is stronger than the evidence around the impact of FOPL on consumer behaviours, such as product identification and consumption. One limitation is that current national dietary intake survey methodology cannot assess the impacts of FOPL on consumption unless surveys capture brand specific data. Although recent data from Chile show significant reductions in sales of foods with the mandatory FOPL implementation (14).

The main challenge in reaching a consensus around any national FOPL policy - beyond getting the policy adopted at all - is the choice of the symbols to be used. Although research shows that interpretive symbols such as warning labels (rather than presenting guideline amounts) are most effective (11, 15–18), there remain many options. Furthermore, the majority of symbols currently in use are either voluntary or industry-led. Additionally, the effectiveness of symbols can be implicated by other health claims on the pack, with a FOPL symbol alone being more effective than FOPL in combination with other health claims (18, 19). There have therefore been calls to standardize the symbols used (16), and implement mandatory policies to increase overall effectiveness (6, 7, 17).

As the food industry has continued to grow, with large multinational corporations dominating the market, their obstruction of public health interventions that may threaten profits has become apparent (20). Additionally, the replication of tactics used by other unhealthy industries (such as tobacco and alcohol) to resist regulation has been well-documented (21).

Multiple frameworks to categorize tactics unhealthy industries use in lobbying have been published (22–25) and results have shown that frequently used industry tactics include: discrediting scientific evidence or formulating evidence through scientists and front groups (21–23, 26), using public relations to inform public opinion (21, 22, 24, 26), promoting alternatives to regulation such as voluntary schemes or pilots (22, 24, 25), amplifying economic importance and impact on industry (21, 22, 26), and threatening legislation (23, 24, 26). Furthermore, industry stakeholders have been found to invest in long term relationship building approaches to exert influence on policymaking (27) whilst silencing those who advocate for healthier diets by discrediting scientists (28).

Health Canada (HC) published the Healthy Eating Strategy (HES) in 2016 outlining a suite of nutrition policies aimed at increasing the healthiness of the Canadian food environment (29). Research on the strategy found that industry stakeholders initiated, and had a greater proportion of interactions with Health Canada (HC) than non-industry stakeholders (22, 30), and attempted to influence policy by framing the debate on diet, promoting deregulation, and promoting alternatives (22). One of the proposed policies included a mandatory FOPL approach (29) where the labelling requirements included a nutrient-specific interpretive symbol that would be required to be displayed on foods that met or exceeded the threshold of 15% of the daily value requirements for nutrients of public health concern (sodium, sugars, and saturated fat) (31).

As part of the strategy approach HC explicitly stated that “*the food environment is a complex and interconnected network of factors and public policy needs to affect multiple parts of the network to affect real change”* (29). The food system in particular has been widely recognized as a complex system (32–34). Complex systems are described as dynamic, with multiple interconnecting components that interact in often random ways (35). Such thinking recognizes that a linear approach – whereby an action results directly in a relatively predictable change may be limited. For example, FOPL policy would not result in everyone choosing to avoid products with FOPL, however it could result in reduced consumption of unhealthy products and lower obesity prevalence. FOPL is one shift in the system that helps it move in the desired direction, health-wise. Although the FOPL policy is intended by HC as one of a range of interventions, a criticism of the policy by some, is that it requires high levels of individual agency – to act on the information to eat healthier foods. By proposing FOPL as a mandatory scheme, the HC policy mitigates these criticisms to some extent, as evidenced by the impact of a similar policy in Chile (12, 36).

The proposed policy underwent two consultations in 2016 and 2018 detailing the FOPL approach and symbol to be used. HC committed to a Transparency and Openness policy (37) where all correspondence and meetings with stakeholders external to public consultations were published in an online database (38). The proposed regulations were published officially in the Canada Gazette, in February 2018 (39), with plans for publication of the final regulations to follow later that year which did not occur, although publication of the final regulations were listed in the Forward Regulatory Plans for 2021–2023 (40) and eventually published in July 2022 (41).

As part of a range of policies to address the complex issue of unhealthy diets in Canada, the FOPL policy is an evidence-based, effective policy that had undergone extensive consultation with stakeholders, yet was stalled for several years. Interactions and consequently influence of stakeholders on policymakers can impact the progression and ultimate implementation of policies. The aim of this study was to analyze stakeholder interactions – through published correspondence and meeting notes – to identify narratives presented by industry and non-industry stakeholders, and their potential role in the policy’s delay.

## Materials and methods

This qualitative study employed framework analysis (42, 43) to analyze correspondence and meeting notes published by Health Canada related to the proposed FOPL policy. The analysis was deepened using a complex systems lens.

### Data selection

Health Canada’s Meetings and Correspondence on Healthy Eating (MCHE) database (44) was developed as part of the Government of Canada’s Regulatory Transparency and Openness policy (45). The MCHE database contains detailed records (hereafter referred to as documents) of all the meetings and correspondence that were shared between stakeholders and Health Canada related to the Healthy Eating Strategy. Documents spanned a time period from 2016, when the Healthy Eating Strategy (HES) was introduced, until 2019 at which point the final regulations had not been introduced and a general election was held (46).

All documents labelled by HC with the subject ‘Front-of-Package Labelling’ were extracted in November 2019 (44); they included meeting notes, presentations, letters, and emails. Duplicate copies of documents (e.g., French versions of English documents or handwritten copies of digital documents), documents that did not refer to FOPL explicitly, and HC publications (e.g., HES report) were excluded from the sample for analysis.

Document date, stakeholder name, and type of meeting (stakeholder- or HC- initiated, as indicated in the MCHE database) were extracted from the database. Stakeholder types were categorised in line with previous research analysing the HES (22, 30) as: ‘industry’ (organisation with a commercial interest, e.g., food companies), ‘non-industry’ (organisation with no commercial interest, e.g., health bodies), or ‘mixed’ stakeholders from the former categories or other organisations.

### Data analysis

Basic quantitative analysis of the documents was conducted in Microsoft Excel to show the quantity of documents submitted from each category and type of documents. Documents were then imported into NVivo 12 (47) to facilitate the framework analysis; this was followed by more in-depth analysis using a complex systems lens.

### Framework analysis

Framework analysis is a systematic methodology commonly used in policy analysis (42, 43, 48). The methodology was developed for use in large-scale policy research, and since adapted for health research (48). It enables the condensation of large volumes of data into a matrix output comprising cells of summarized data (48). Strengths of this methodology in policy research include the use of pre-set aims and objectives identified from the outset, and the ability to include *a priori* issues while remaining grounded in the data (43). Additionally, the ease of collaboration during the analysis increases inter-rater reliability (42). The five steps of framework analysis include: familiarization, identification of the thematic framework, indexing, charting, and mapping and interpretation.

In the familiarization step, the first full read through of the dataset is used to generate a log of key ideas and themes in the data through an inductive approach. Next these themes are developed, and are incorporated with *a priori* themes, in an iterative process that results in the identification of the thematic framework. In the indexing step, the data is coded into the thematic framework topics, with relevant lines of texts being selected as a ‘code’ and assigned to a topic. In the charting steps these codes are grouped into the topics, collectively reflecting certain views and experiences. The final mapping and interpretation step involves creating a framework matrix with the thematic framework topics. During this process, the findings are analyzed and reviewed to identify the key themes in the data.

The first author completed the familiarization log, and then the thematic framework and resulting charts were presented to co-authors to validate the identified themes and interpret them collaboratively. *A priori* themes from the literature including industry tactics, economic and opportunity cost, and public health approaches were discussed and incorporated where appropriate (22, 23, 49, 50). The thematic framework charts provided a summary of the directly observable ideas (51), and overarching themes across all the thematic framework charts were found during their analysis. These findings are presented in the thematic analysis results. In the final stage of mapping and interpretation, the analysis was deepened by incorporating a complex system lens.

### Complex systems analysis

Complex systems research has been widely used in other disciplines, but only more recently gained traction in health research (35). A complex system is a dynamic system of interacting components, including actors, who could be individuals, groups or organizations (52). The boundaries around the system are in themselves dynamic and changing (52). However, in order to make the system comprehensible they are artificially imposed, and hence must be placed appropriately to ensure the full impacts of a policy on the system are captured (53).

To describe the changes within a complex system, the guidance created by the United Kingdom’s National Institute of Health Research School of Public Health Research (SPHR) summarized the terminology used, and is presented in Table 1 with a FOPL specific example for each term (52).

**Table 1:** Terms to describe changes in a complex system by Egan et al. (52) and a FOPL example to illustrate.

Terms for complex system (52)	Front-of-package labeling example
System cohesion	Are stakeholders aligned and in agreement that FOPL is an appropriate policy to improve nutritional choices?
Stable and unstable systems	Would the FOPL policy have any impact on the system or will the change be absorbed and lead to no overall change in food choices?
Non-linearity	Could introducing the FOPL lead to a significant change such as transforming consumer expectations of product composition and thus change the food system disproportionately?
Trade-offs and choices	By introducing FOPL, what other policy options were not introduced?
Unintended consequences	What unintended consequences occur? e.g. can the FOPL result in consumers picking less nutritious foods unintentionally?
Emergence (scope)	To what extent is the FOPL policy scope expanded to apply to a greater ‘system map’ such as digital innovations or restricted to limited products?
Adaptation	What changes will be made to how the system works as a result of the policy? Will industry adapt by reformulating to avoid having to apply FOPL to their products?
Spill-over/displacement	Could the policy move the issue to another area rather than resolving it, e.g., by applying FOPL to certain products will it result in the consumption of alternative unhealthy products?
Feedback	Could the policy result in an accelerated action where it creates a positive or negative feedback loop, e.g., can the FOPL change consumer taste demand for further reduced sugar products?

The benefits of taking a complex systems approach allows easier conceptualization of the many factors at play in a given issue and better anticipation of unexpected and counterintuitive consequences (35). The majority of work around complex systems remains theoretical, with limited application in generating evidence or effective policy (32). In order to make a systems approach more accessible and effective, the SPHR created guidance on how to generate research that takes complexity into account and to evaluate interventions appropriately (52, 54). Given that FOPL is one intervention in the ‘system’ of diet-related poor health, and the explicit reference to complex systems in the HES (29), it is valuable to examine it with a complex systems lens.

A novel approach was employed by this study to adapt the Framework Analysis methodology by incorporating the SPHR systems terms (52) in the final mapping and interpretation stage of analysis. To create the framework matrix, the thematic framework topics were cross-tabulated with the complex systems terms (presented in Table 1). This resulted in the cells summarizing references in the data where complex system concepts were being directly or indirectly expressed. Further themes from this extended systems analysis are presented in framework analysis results.

## Results

### Overview

**Figure 1:**
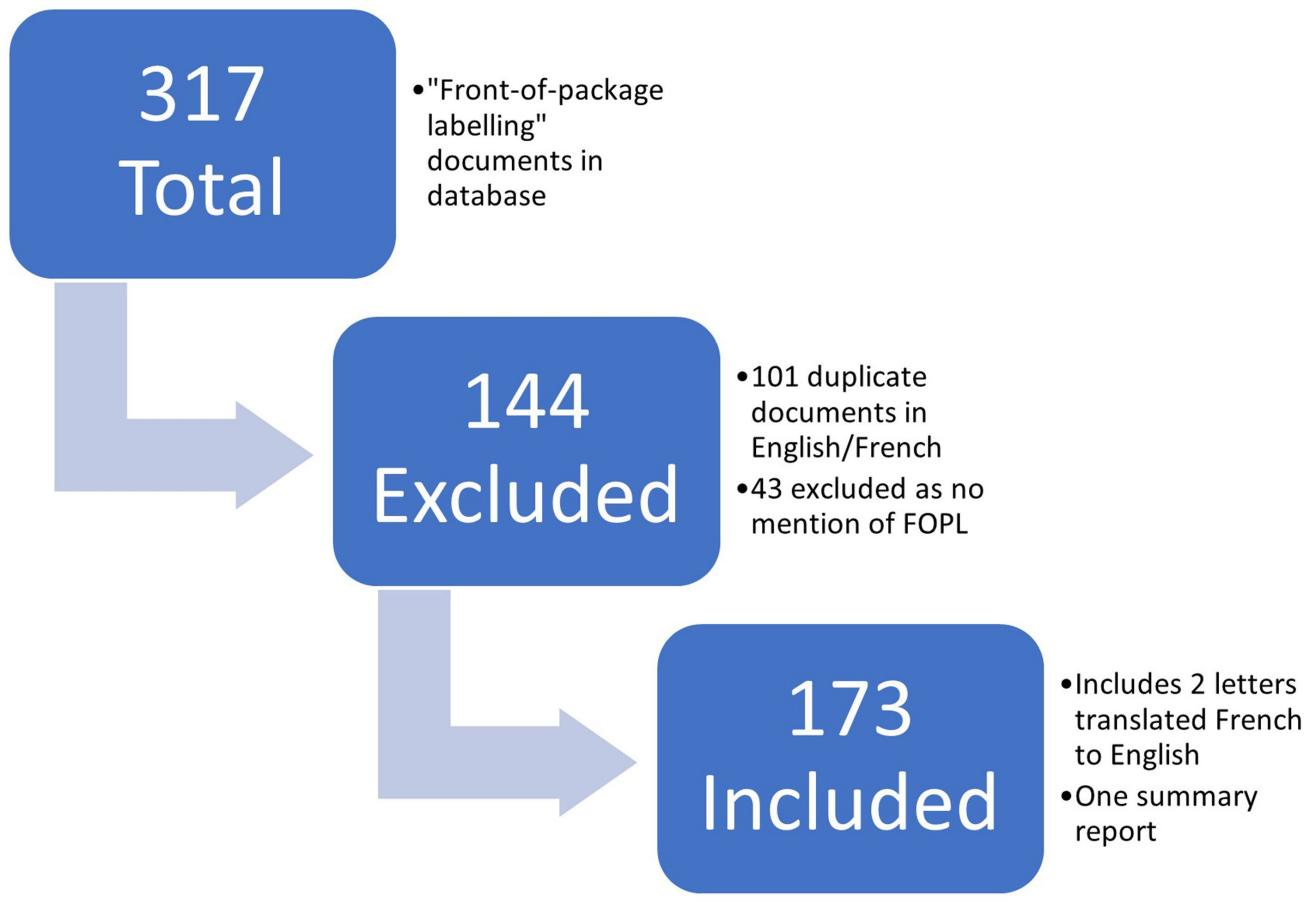
Flowchart to show documents included in the analysis.

There were 317 documents labelled by Health Canada (HC) under the topic “Front-of-package labelling” extracted from the Meetings and Correspondence on Healthy Eating (MCHE) database. Hundred and seventy-three were included in the final analysis after exclusions (such as French or handwritten duplicates) as presented in Figure 1. Documents covered the time-period from December 2016 to June 2019.

Most documents were recorded by HC as being stakeholder-initiated interactions (*n* = 144, 83.2%); 6.9% (*n* = 12) were HC-initiated, and the remaining interactions were initiated by both HC and stakeholders (*n* = 17, 9.8%). When categorized by stakeholder type, almost two thirds of all documents (*n* = 108, 62.4%) were from industry stakeholders. A breakdown of documents by stakeholder type is presented in Table 2.

**Table 2:** Stakeholder types.

Stakeholder type	Description	Total (%)
Industry	Organization with a commercial interest	108 (62.4%)
Non-industry	Organization with no commercial interest	37 (21.4%)
Mixed	Industry and non-industry stakeholders together Other organizations	28 (16.2%)
Total		173 (100%)

As per the framework analysis steps, after familiarization, eight topics were identified in the Thematic Framework (column one in Table 3) with three overarching themes emerging from analysis across the topics.

**Table 3:** Framework matrix.

		Complex system terms								
		System cohesion	Stable and unstable systems	Non-linearity	Trade-offs and choices	Unintended consequences	Emergence (scope)	Adaptation	Spill-over/displacement	Feedback
Thematic Framework Topics	Industry’s role as a stakeholder	**-Industry want FOPL aligned with trade partners**	**Unstable system where FOPL limits Canada’s potential -Trade pressure on Canadian market**	**-FOPL can have disproportionate impact on Canada’s potential**	-**Economic *vs.* societal costs** **-Regulatory *vs.* business cost**	**-Reformulation may not improve product**		-Business to adapt to healthier goals. -Motivate reformulation to avoid FOP		
	Industry’s engagement in policy development	-All sectors have a role to play in food industry	-Uneven playing field for industry							
	Concerns about the policy		**-FOPL can have disproportionate impact on Canada’s potential**			-Disproportionate targeting of nutrient dense products	-Widened to consider digital approach **-Widened to multi-factorial solutions and promotion of healthy diets rather than nutrients** **-Widen to apply to all foods**	-Move to digital interface **-Negative adaptation to additional calories in poorly reformulated products**	- Displacement from healthy diet to reduced healthy calories (e.g., less diary consumption) - Additional calories in poorly reformulated products	
	Consumers response to the policy	-Industry ‘opposed to... Transparency’			-Deprioritize educating consumers	**-Consumers cannot distinguish healthier products** -Undermine public trust -Inappropriate use of safety symbol -Conflicting health claims -Health halo **-Uneven playing field for industry**	**-Narrowed to NFt**	-Positive adaptation where it leads to change in consumer behavior		-Positive loop where consumers and manufacturers improve products in a mutually reinforcing way
	Industry perception of a weak evidence base for the policy	**-FOPL does not align with agri-food industry system**			-**Business Investment *vs* health**	-Multiple clashing symbols on products -Widespread application leading to insensitivity	-Widened to say FOPL will not resolve obesity **-Widened to health rather than behavior change**	-Positive adaptation where it leads to change	-Discouraging nutritious foods	-Positive feedback loop where reduced beverage calories lead to further reductions	
	Global policy comparisons	**-Industry want FOPL aligned with trade partners.** -Non-industry note a global move to improve food offering	**-Concerns about potential reputational damage to Canadian products and impact on trade**	**-Reputational damage to Canadian products -Undermine trust in food industry**						
	Practicalities of policy implementation	-Cohesive approach between FOP, serving size, and NFt			-Cost of delays in policy implementation on industry vs. health	**-Exemptions lead to uneven playing field** -Including HC name could be seen as endorsement. -Misleading nutritional health advice **-If threshold cannot be reached will disincentivize manufacturers** -Uneven playing field with negative brand power for pre-packaged foods	-Widened to future trends and retail environment -Widened to non-pre-packaged foods. **-Widened to apply to all packaged food and beverage** -Narrowed to serving size	**-Negative adaptation if cannot meet thresholds to reformulate may increase sugar/sodium/fat to meet competitive and consumer taste preference**		
	The Front of package symbol	**-Using a current industry FOPL symbol promotes system cohesion**				-Confusion with a safety symbol				

Blank squares indicate there were no explicit/implicit reference between the thematic framework topic and system term within the data. Bold type indicates references from a greater number of stakeholders.

### Thematic analysis

Familiarization with the data found no apparent changes in the narrative over time, thus all the documents were analyzed collectively. During this first step a log of ideas were kept and eight key topic groupings emerged. *A priori* issues such as industry tactics, economic and opportunity cost, and public health approaches were also incorporated as they were found to be relevant by the authors (22, 23, 49, 50), and the thematic framework was agreed by authors in an iterative process. A detailed breakdown of each the eight topics and the corresponding codes can be found in Supplementary Table S1. In the indexing step the codes were applied to all documents, and then the codes were pulled from the documents and charted under the applicable topic. Analysis of the codes across all the topics using inductive reasoning found three overarching themes that were present: (1) industry controlling the agenda, (2) Industry questioning the evidence base of the policy and impact on trade and competitiveness, and (3) Industry dismissing the policy. The three key themes are elaborated on below.

### Theme 1: Industry controlling the agenda

Although industry actors refer to themselves in the FOPL consultation process as ‘stakeholders’, there is a clear sense of them attempting to lead the decision making: they appeared to expect to be able to change previous decisions taken in the policy development process. This was most apparent in discussions on the selected FOPL symbol, where even after being presented with the evidence base and criteria for the proposed symbol, industry stakeholders continued to reject it:

*“During the Meeting food industry participants specifically stated for the record that there was no agreement to design principles and called for a robust and inclusive dialogue to take place to develop appropriate, scientific and evidence-based principles for FOP labelling. It is therefore inappropriate for Health Canada to impose the four design criteria outlined in the Letter.”*

(Industry stakeholder, 04/10/2017)

Industry stakeholders implied they have a leadership role compared to other stakeholders in the decision making due to a perceived unique insight into the FOPL policy and its impacts. There were also many references to the benefits they provide to the economy and in employment.

“*Food industry has an immense amount of knowledge and data that no other stakeholder can provide.*”

(Industry stakeholder, 26/05/2017).

As an alternative to regulation, industry stakeholders promoted the use of their own FOPL symbols or labels and highlighted that some of the symbols were already in widespread use. Voluntary schemes and pilots were also promoted as an alternative.

*“Pilot and evaluate the voluntary introduction of a neutral fact-based front-of-package system on a sample of foods and compare its results to existing programs.”*

(Industry stakeholder, 26/05/2017)

Finally, rather than considering FOPL, some industry stakeholders focused elsewhere, and reported slow regulatory processes were the limiting factor in reformulating and improving products and should consequently be corrected first.

*“The Canadian food and beverage industry continues to face challenges with timely regulatory approvals and costs for reformulation and innovation. Because of outdated regulations, it takes far longer to bring new and reformulated products to market in Canada than in other countries. Health Canada and the Canadian Food Inspection Agency must address lagging regulatory modernization quickly – before imposing new regulations*.*”* (Industry stakeholder, 18/09/2017).

### Theme 2: Industry questioning the evidence base of the policy and impact on trade and competitiveness

The policy was questioned by industry actors in terms of whether the evidence supporting FOPL was sufficient, and whether the economic impacts of the policy was justifiable. Discussions around the economic impact and potential impact on trade and competitiveness were from a trans-national corporation perspective, with little or no mention of small business. While many industry organizations stated they supported evidence-based policies to improve health outcomes, they did not acknowledge the evidence and consumer and market research summarized from the preceding consultation (55) that specifically documented the evidence and need for a FOPL, rather than the aim of this consultation which was to focus on the final form the FOPL would take.

“*there is no defendable evidence which supports a positive correlation between a change in consumer choices and improved health resulting from warning labels on the front of pack*” (Industry stakeholder, 03/07/2018).

Although there was some opposition to the demand for further evidence and the validity of the FOPL policy by non-industry stakeholders, it was minimal compared to the industry stakeholder voice. In particular, the FOPL policy introduced in Chile (similar to the proposed HC FOPL) was mentioned by many, though only non-industry stakeholders referred to the positive impacts of the Chilean policy in changing consumer behavior and driving reformulation. Industry stakeholders argued the impact was short lived, and the evidence on its success was weak. Significant focus was paid to previous opposition of the scheme by a Canadian government official at trade meetings, and to the lack of longer-term evidence.

“*Canada challenged Chile regarding their similar FOP initiative within WTO Technical Barrier to Trade (TBT) meetings in 2015 and 2016. Noting that Chile’s requirements deviated from international standards… were not based on science and were more trade-restrictive than necessary. It is inexplicable that Health Canada would now forge ahead with its own divergent FOP initiative.”* (Industry stakeholder, 04/10/2017).

Industry stakeholders contributed to the evidence base by submitting industry-commissioned literature reviews describing specific nutrient evidence and arguing for exemptions. They also presented other industry-commissioned research, carried out to gage consumer opinions on the FOPL symbol, and its impact. However, non-industry stakeholders argued the methodology used in this research was poor, as there was only theoretical, and not functional testing of participants preferences, and hence was of low quality compared to the extensive market research conducted by Health Canada in their preceding consultation (55).

*“Clarification is needed on the methodology used by* [redacted] *for their survey. There are important limitations inherent to public opinion research.” (Mixed stakeholder summary report, 19/09/2017).*

Industry stakeholders also criticized the FOPL cost benefit analysis published by HC as “*lack[ing] balance and nuance*” (Industry stakeholder, 30/05/2018) by not accounting for future regulatory changes. Although the HC response to another industry stakeholder reported “*both costs and benefits were calculated using a conservative approach, with the intention of costs being overestimated and benefits underestimated*.” (Non-industry stakeholder, 04/06/2018).

Industry stakeholders reporting feeling “*initiatives unfairly target the food processing industry*” (*Mixed stakeholder summary report, 19/09/2017*) and had concerns about the impact the policy would have on their business and ability to trade. With regards to individual businesses, they reported concerns about the economic cost of the combined food policy changes proposed as part of the HES, with stakeholders arguing they would need to divest from other areas, such as innovation, to compensate.

“*Given the magnitude of the costs associated with food labelling proposals, industry’s ability to invest in equipment, R&D or new product development will be impacted as innovation capital is reassigned to regulatory compliance.”* (Industry stakeholder, 14/05/2018).

The FOPL policy was perceived by the industry sector to create “*an uncertain and onerous regulatory environment that will decrease investment in the food sector in Canada*” (Industry stakeholder, 04/10/2018) and risk changing the way the food industry was viewed: “*Label foods like foods, and not like drugs, alcohol or tobacco”* (Industry stakeholder, 24/05/2018).

Concerns about the impact on international trade were dominant among industry stakeholders. Using a different approach to trade partners, or using a different FOPL symbol to that used by many trans-national corporations, were perceived to cause a negative reputational impact on Canadian goods. Industry stakeholders reported the FOPL policy may “*be seen by other countries as a non-tariff barrier to trade for their imports into Canada*” (Industry stakeholder, 29/06/2018) and would subsequently have a significant effect on the economy.

There was limited discussion on the impact of the policy on behavior change and health outcomes, with the exception of one non-industry stakeholder:

*“As you and your officials weigh the potential cost to industry of new FOP labelling regulations against the known costs associated with chronic over-consumption of these nutrients on Canada’s already-fatigued health care system, we encourage you to place the emphasis on Canadian’s health and wellbeing*” (Non-industry stakeholder, 11/12/2017).

### Theme 3: Industry dismissing the policy

Industry stakeholders dismissed both the need for the policy and its effectiveness, if it were introduced. This theme also covered the technical aspects of the FOPL policy such as what products it applies to and the proposed nutrient thresholds for triggering a warning label.

Industry stakeholders denied the need for a regulated FOPL symbol by referring to initiatives they already had in place or were planning to introduce. Many industry stakeholders argued they already had a FOPL symbol on their products, and prompted HC to adopt an industry FOPL symbol, arguing that it would be less trade restrictive.

*“*[Industry stakeholder] *indicated that fact-based FOP labelling has already been implemented, or proposed, in many countries on a voluntary basis…only Chile has a mandatory interpretive system*.” *(Mixed stakeholder summary report, 19/09/2017).*

Additionally, many industry stakeholders illustrated their commitment to health policies by referring to specific nutrient efforts such as work to reduce sodium in products, whilst others referred to broader campaigns to reduce calories in beverages or signing up to a global marketing to children code. Only one industry stakeholder referred to work to directly address the nutrients covered by the FOPL policy.

“*I know one of the key objectives of the HES it to try and get industry to reformulate. As you know* [Industry stakeholder] *has been working for the past five years to decrease the amount of sugar, salt and fat in our products and has dedicated significant resources towards doing so*.” (Industry stakeholder, 07/07/2017).

Industry stakeholders questioned the policy effectiveness at changing product offerings, encouraging manufacturers to reformulate, or at changing consumers purchasing patterns:

*“How do you know that labels will be effective, given that there is no convincing evidence in the literature…What evidence was there that indicates that labels will change consumer behavior that leads to better health outcomes?”* (Industry stakeholder, 14/06/2018).

Many stakeholders (industry and non-industry) felt that the application of the FOPL policy only to products with Nutritional Facts tables (NFts), and not all food products in retail settings, would result in an unsuccessful policy. This was acknowledged by HC as a gap that they hoped to address in future. It was also argued by industry stakeholders that the proposed set thresholds of 50 g for the FOPL policy would disincentivize reformulation by manufacturers.

“*A 50g threshold removes any relationship between FOP labelling, the NFt, and the serving size. It also removes incentive for companies to reformulate their products*” (Industry stakeholder, 04/10/2017).

They also argued that even if they were to reformulate, it would not necessarily improve the nutritional profile of the product.

“*Reformulation to reduce or replace sugar content in foods may not improve their nutrition profiles or reduce caloric contents, as sugars will likely be replaced with refined starches and maltodextrins*” (Industry stakeholder, 30/05/2018).

Furthermore, the FOPL symbol was presented by industry stakeholders as being confusing for consumers, and that the focus should be on education instead. Ways in which the symbol was considered confusing included if it displayed other conflicting nutritional claims such as ‘*lower in*’ alongside the FOPL warning, or was a symbol typically used in warning settings (e.g., hazard sign).

*“As outlined in our original proposal, and further expanded upon in the attached, octagons, triangles and exclamation marks are regulated for the purposes of denoting a safety hazard. Their use on food products would inappropriately suggest a food safety risk, and their prevalence on foods could also undermine their effectiveness as safety warnings*.” (Industry stakeholder, 18/09/2017).

Educating consumers was considered important by all stakeholders, although industry-stakeholders tended to prioritize this above the FOPL policy, rather than alongside it, which was the approach taken by most non-industry stakeholders. Additionally, industry stakeholders also offered to contribute to the organization and financing of such initiatives.

*“Moreover, education is the first and most effective tool to change behavior — and that is exactly what the Government is not doing: educating the public.”* (Industry stakeholder, 18/09/2017).

### Framework analysis

The framework matrix was constructed by cross-tabulating the thematic framework with characteristics of a complex system. Where there was an implicit or explicit reference to a system term in each theme, it was recorded in the matrix and is presented in Table 3. Although there were some references to all the system terms, those cited more often and by a greater number of stakeholders are grouped and presented in the findings (highlighted in bold in Table 3). The groupings in order of dominance were as follows: (1) system cohesion, stable/unstable systems, and non-linearity (2) trade-offs and unintended consequences, (3) emergence and adaptation, and (4) spill-over/displacement and feedback. Due to the majority of interactions being from industry stakeholders, the majority of references to system terms are theirs.

#### System cohesion, stable/unstable systems, and non-linearity

Industry and non-industry stakeholders had markedly different perspectives on how cohesive, or aligned, the system was due to the fundamental antagonism between profiting from the sale of unhealthy products and the health benefits of minimizing consumption of such products. However, they both agreed that the policy has the potential to initiate change rather than just being absorbed without impact, hence the system was unstable.

Many industry stakeholders described the policy as specifically disadvantaging their interests, creating an uneven playing field in the sector, restricting potential sales and growth, and causing a loss of trade. In particular, they described the agri-food industry as non-cohesive and competitive internationally and that applying FOPL on Canadian products would have a detrimental impact on them. Additionally, changes in trade with the United States (US) through the impending North American Free Trade Agreement (NAFTA) negotiations, and tax and tariff differences also indicated the system was non-cohesive. They identified a non-linear, and disproportionate response to the FOPL symbol as having the potential to damage public trust, reputation of Canadian goods, and the agri-food industry’s future potential. Contrastingly, the unanimous promotion of current industry FOPL policies to mitigate trade risk, depicts a comparatively more cohesive sector that can be aligned to achieve the policy aims.

*“Putting stop signs on Canadian made food products will undermine confidence in our agriculture and food industry in Canada and abroad. This approach would most likely result in great harm to the industry’s reputation, undermining public trust in Canadian food and industry’s continued efforts to capture emerging markets, both domestically and internationally*” (Industry stakeholder, 09/03/2017).

Non-industry stakeholders’ voices, including HC, were quieter in comparison, but described a cohesive system where stakeholders could work together to improve the food offering, of which FOPL is one part of the policy approach.

*“The Healthy Eating Strategy provides an opportunity for the food industry to adapt business practices to align with healthy eating goals while being economically successful. This strategy, which is supported by regulations, will provide the agri-food sector an important opportunity to grow, develop, and market healthier foods.”* (Non-industry stakeholder, 04/06/2018).

#### Trade-offs and unintended consequences

Trade-offs as a result of the policy were considered in terms of economic cost and opportunity cost. Regarding the economic impact of the policy, industry stakeholders felt the short-term costs to industry were underestimated and that the long-term costs would be substantial, due to the potential impact on trade. In comparison, non-industry stakeholders argued that the economic trade-off, whatever the extent, was limited compared to the opportunity cost of improving health outcomes.

*“The truth of the matter is that while these spokespeople seem to be engaging in economic fearmongering and getting up in arms about logos intended to reveal more clearly the true nutritional value of many food products on the market, overweight and chronic diseases associated with unhealthy eating are taking a heavy toll on our society*.” (non-industry stakeholder, 08/08/2017).

A second issue was in the timing of policy implementation where industry stakeholders requested a delay in enforcing the policy to allow industry time to absorb the costs more gradually. This was rejected by HC as a trade-off between delaying for industry benefit vs. delays to societal benefit.

“*Giving industry an extra year to implement changes would delay the benefits to health, which also has significant economic implications*.” (non-industry stakeholder, 17/12/2017).

A third trade-off between the aims of educating consumers or changing behavior with FOPL was only explored by industry stakeholders, as non-industry stakeholder considered them complementary initiatives.

*“If the goal is to influence consumer purchasing behavior, rather than education and information, Health Canada must demonstrate the efficacy of its approach in light of the trade-restrictive nature of its proposal compared to other equally effective approaches.” (industry stakeholder, 18/09/2017).*

Industry stakeholders raised several potential unintended consequences. Varied reasons for the FOPL symbol being potentially misinterpreted were given, including whether exemptions can cause consumer confusion. As the policy proposed to include the FOPL symbol only on pre-packaged products with a Nutrition Fact tables (NFt), both stakeholder groups argued exempted foods not subject to the NFt policy, and hence exempted from FOPL, could be misinterpreted as healthier, by virtue of not displaying the symbol.

*“Not only will the food industry have to bear the costs of the change alone, but many of its products will have a logo for those that exceed the standards, while retail and bulk foods are not subject to regulation... For example, consumers could estimate that lasagna prepared at retail and sold at the refrigerated counter is superior in terms of nutritional quality.”* (Industry stakeholder, 29/11/2017).

Furthermore, industry stakeholders argued the policy may result in less healthy diets if nutrient dense products are included.

“*A number of participants expressed concern that warning symbols do not discriminate between nutrient-dense foods and others. There could be unintended consequences, such as children under 2 years old being fed low fat milk*.” (Mixed stakeholder summary report, 18/08/2017).

Finally, unintended consequences of the symbol used included concerns by industry stakeholders about the use of common safety symbols as deeming food unsafe, or the inclusion of HC’s name within the symbol as being considered an endorsement of the product rather than mark of authority.

#### Emergence (scope) and adaptation

The FOPL policy proposed by HC had clear goals that were set out in the consultation guidance. Using the FOPL policy proposed by HC to inform the scope of the system, however, it becomes apparent some stakeholders widened or narrowed the scope of the policy.

Proposed ways to narrow the policy by industry stakeholders included exempting certain products such as those with small serving sizes. Furthermore, there were calls for specific exemptions such as dairy, fruit juices, cranberries, and others.

*“In addition, Health Canada is not giving adequate consideration to the impact FOPL will have on confections, which self-regulate via portion control. Many confections are offered in individually wrapped portions, which are consumed as occasional treats, rather than meal supplements or components. If Health Canada proceeds with FOPL, an exemption should be granted for confection products“* (Industry stakeholder, 30/05/2018).

Some industry and non-industry stakeholders promoted applying the FOPL policy to a wider range of foods, whilst other industry stakeholders argued this in itself would make it less effective.

*“Consumers who will be asked to choose from a majority of products displaying logo could develop insensitivity and override these warnings”* (Industry stakeholder, 29/11/2017).

Other suggestions of widening the policy scope included considering larger health outcomes such as diets overall and obesity trends rather than quantifiable behavior changes such as selecting foods without FOPL labels. Future digital innovations and consumer demands were also suggested as a way the policy could be broadened.

“*By the time the regulations come into force, our members anticipate significant technology driven changes to the way consumers shop for food*,” (Industry stakeholder, 18/09/2017).

The HC FOPL policy’s stated aims were to change consumer behavior and result in food industry adaption through reformulation. This positive adaptation was discussed exclusively by non-industry stakeholders, and one industry stakeholder who was an anomaly in the responses. In comparison, other industry stakeholders argued manufacturers who could not meet the thresholds would instead adapt by developing a less healthy products (e.g., with increased sugar content) to compete with other products and increase consumer demand.

*“sugar, sodium, or saturated fat content of products could unfortunately increase to meet competitive and consumer taste preferences”* (Industry stakeholder, 20/04/2018).

#### Spill-over/displacement and feedback loops

There were much fewer references to displacement and feedback loops, and the few that were found related to other system terms too. Discussion of displacement focused on detrimental impact on nutritional intake if consumers shifted to low-fat dairy products or increased nutrient dense food consumption (which were also considered as unintended consequences of the policy). A further example given of displacement was increasing the use of sugar in reformulated products to improve taste when sodium and fat were reduced.

There were two references to positive feedback loops, the first referring to an ongoing reduction in beverage calories in Canada, and the second recognizing the intended policy outcome of reformulation and healthy product identification. Of note, the latter, depicted in the quote below, was an anomaly response and this view was not shared by other industry stakeholders.

“*FOPL is a promising intervention that can make healthier food choices easier by nudging both consumers & manufacturers in a mutually reinforcing way”* (Industry stakeholder, 13/07/2017).

## Discussion

Between 2016 to 2019, 173 individual documents were submitted to the MCHE database discussing Front-of-Package Labelling. The majority of documents were stakeholder-initiated, and almost two-thirds of all documents were from industry stakeholders, indicating that the discourse around this policy was dominated by industry viewpoints.

Using framework analysis to qualitatively analyze the documents, a thematic framework emerged with eight topics (detailed in Supplementary Table S1). Analysis of the thematic framework yielded three overarching themes: industry controlling the agenda and resisting regulation; questioning the evidence base of the policy evidence and impact on trade and competitiveness; and dismissing the policy and the need for it. The analysis was deepened using a complex systems lens, by cross-tabulating the thematic framework topics with characteristics of a complex system (see Table 3). This process highlighted that industry and non-industry stakeholders held markedly different perspectives on system cohesion, however, both agreed that it was an unstable system. Economic cost and opportunity cost of the policy were seen as the main trade-offs, and concerns of unintended consequences around misinterpretation of the symbol were reported. With regards to emergence, some stakeholders argued for a wider policy scope, whilst others requested a narrower approach through exemptions. Finally, limited discussion of adaptation and spill-over/displacement as a result of the policy was referred to.

Our study identified that industry stakeholders opposed the proposed FOPL policy whilst promoting multiple alternatives, including industry promoted FOPL initiatives. This is reflective of the global picture where the majority of FOPL policies are voluntarily applied, and in some cases may be FOPL symbols preferred by industry (8, 56). Globally, Latin America has had the most success at implementing mandatory policies, the majority of which are black and white octagonal ‘warning or high in’ symbols (8). In comparison, countries in the European continent and Australasia almost all have voluntary policies in place using summary systems, rather than nutrient specific systems (8).

The shift to individualist framing of the FOPL policy outcomes, where industry stakeholders anticipated that consumers will be confused and require education, detracted from the structural changes needed to respond to diet-related chronic diseases as a population health issue (57, 58). Employing complex system theory helps to interpret some of the industry claims and brings to the forefront the lack of cohesive vision on policies, due to misaligned goals between industry and non-industry stakeholders. The industry focus on less effective downstream interventions (50) and resistance to more effective interventions or regulation (59, 60) through juxtaposing narratives becomes more apparent through a complex system lens, enabling policymakers to address such arguments more promptly and robustly.

The ‘policy cacophony’ created by the multitude of policy options including industry-promoted symbols, or educational programs inhibits progress by drowning out concerted, coherent efforts (61) and delaying policy implementation (62). Our study reported multiple instances of competing policy recommendations, such as requests for exemptions, juxtaposed with requests for universal application of FOPL to all retail products. Similarly, research published by Health Canada was ignored, while calls for more market research were made simultaneously. Therefore, it is plausible that industry stakeholder’s opposition resulted in delays, either directly through lack of agreement on the symbol to be used, or indirectly through a prolonged stakeholder consultation phase, in which the implementation of the HC policy remains to be implemented. The main findings of this analysis resonate with other studies conducted on stakeholder interactions in relation to the HES (22, 30), namely that a large proportion of interactions are industry-stakeholder driven, and that these stakeholders frame the narrative to fit their interests. The ‘louder’ industry voice which accounted for 62.4% of all documents further highlighted the limited, opposing public health voice. Food industry corporate political activity to influence policy has been documented in a range of countries (49), over long time periods (27), and more active lobbying has been found where there is a greater potential pay-off for the organization (63). In comparison, Stuckler et al. argue that public health professionals are slow to respond to nutritional threats due to discomfort in tackling powerful companies’ vested interests (20, 64). Additionally, there tends to be a lack of coordinated effort and resource by health professionals to proactively frame public health arguments to balance the food industry lobbying cacophony (61, 65).

Additionally, many previously documented industry tactics were prevalent in the material examined in this study. Tactics were wide ranging, including resisting regulation through the promotion of voluntary schemes and pilots (20, 22, 25, 62), and promoting industry FOPL policies over those proposed by HC (20, 22, 59). Additionally, there was one instance of industry stakeholders volunteering to fund educational campaigns as an alternative to regulation (59, 62). Industry stakeholders also discredited scientific evidence (23, 26) and denied the impact of FOPL policy on health outcomes, however, they promoted industry funded research of questionable rigor (23). The amplification of economic importance and impact was apparent in many documents when considering the impact on trade (21, 22). Of note, there was no mention of the impact on small businesses, indicating it was predominately the views of large trans-national corporations that were being represented.

Whilst this study embeds complex systems theory into the methodology, similarly to the majority of the literature on obesity as a complex system, the findings do not directly identify where solutions could be applied (66). However, unique insight into industry stakeholders’ consultation responses gained through the complex systems lens allows policymakers to manage unrealistic linear arguments, whilst accounting for valid concerns about the impact of the policy on the system. The focus of industry stakeholders on the lack of cohesion in vision is fitting, as it demonstrates the inherent misalignment of goals between health policies and the processed food industry that is well documented. This mis-alignment has led to calls to manage industry stakeholders appropriately in the policy process (20, 23, 64).

Although HC’s Transparency and Openness policy provides a unique insight into the disparity between industry and non-industry activity, the dominance of industry stakeholders shows a more structured engagement approach is needed by policymakers to ensure proportionate representation of all stakeholders in the policy process. A framework created by the World Health Organisation (WHO) with guidance on engagement of stakeholders (67) was criticized as insufficient (68, 69); however, the principles of engagement provide a starting point for policymakers to consider the need, and extent of engagement of industry stakeholders in specific aspects of policy development. Taking a nuanced approach relevant to the healthy food policy being discussed can promote transparency and reduce delays to the implementation of effective, evidence-based public health policies.

### Strengths and limitations

Vast amounts of publicly available data were obtained and analyzed in this study as a result of the landmark Transparency and Openness Framework adopted by HC. The MCHE database provided a unique opportunity to access uncensored primary data from stakeholders discussing the development of the FOPL policy over a period of three consecutive years. The thorough database included copies of all emails and letters as well as notes from meetings. Whilst the letters and emails were very informative, one of the limitations was the variability in how detailed meeting notes were. Generally, while they adequately recorded the contents of the conversation, they did not tend to convey the views held by stakeholders, therefore the analysis and interpretation of meetings was limited to the contents of the meeting notes, that at times were very brief. The database only recorded direct stakeholder interactions with HC, and hence other lobbying activities (e.g., with politicians or other government departments) and other opportunities (e.g., donations or the use of third parties) were not explored. Additionally, interactions between HC and individuals representing themselves were excluded from the database, and this may have underestimated the number of non-industry stakeholder documents (e.g., academic experts), but the number of these interactions is likely to have been limited in comparison to the volume of industry documents.

A major strength of this work was the use of framework analysis, a validated qualitative method for use in policy analysis, which allowed for a systematic approach to manage the large dataset. The methodology allowed for an inductive discovery of the thematic framework, that was complemented by integrating *a priori* themes. Allowing researchers to develop the key themes collaboratively increased inter-rater reliability of the analysis. Additionally, the incorporation of a complex systems lens deepened the analysis by including implicit references made by stakeholders rather than just explicit references in the data. The analysis was limited to the data consultation period, and hence we are unable to analyze the impact of the lobbying on further policy development since this time. Although delayed, publication of the final regulations was listed in the Forward Regulatory Plans for 2021–2023, and were eventually finalized in Canada Gazette 2 in July 2022 (41), but we were unable to quantify what extent this was influenced by stakeholder lobbying or other political factors.

### Conclusion

The Front-Of-Package Labelling policy (FOPL) proposed by Health Canada (HC) is an evidence-informed policy introduced as part of a range of policies in the Healthy Eating Strategy. Analyzing stakeholder interactions through the Meetings and Correspondence on the Healthy Eating database identified differing perspectives between industry and non-industry stakeholders due to misaligned goals. The Transparency and Openness policy by HC allowed greater insight to the consultation process and should be continued and more widely applied in future policies.

The insights of this study can be widely applied, as many of the industry stakeholders are global actors or have shared industry tactics globally. There was strong lobbing for voluntary FOPL policies that industry have chosen, or for the delay of mandatory policy implementation. Continuing to focus on the ever growing evidence-base of the effectiveness of mandatory FOPL and in selecting the symbol for the policy is key to ensuring an effective approach is applied more broadly in other jurisdictions. These data can also help support efforts to ensure the implementation of other healthy food policies.

Industry stakeholders with a vested interest against healthy food policies employed many tactics in an attempt to delay, alter, or prevent policy implementation and hence require careful consideration. Policymakers need to set out their principles of engagement in advance to identify appropriate engagement points for all stakeholders in the policy development process, and proactively ensure proportional representation of all stakeholders (including active enablement of less well-resourced, non-industry stakeholders).

Ultimately, understanding delaying tactics in the policy process can provide lessons for future health-related policies and could ensure that evidence-based health policies such as FOPL are implemented.

## Data availability statement

Source data is available on the Health Canada Meetings and Correspondence on Healthy Eating database. Data available at: https://www.canada.ca/en/services/health/food-nutrition/healthy-eating/meetings-correspondence.html

## Author contributions

AJ and MRL conceptualized the study. AJ, MRL, and NS designed the methodology. AJ and CM completed the analysis with input from all authors. AJ drafted the manuscript. All authors reviewed, edited, and approved the final manuscript.

## Funding

This study was supported by the Canadian Institutes of Health Research (CIHR) grant 201503-MOP142300 (MRL) and CIHR Frederick Banting and Charles Best Canada Graduate Scholarship Doctoral Awards (CGS-D) (CM).

## Conflict of interest

The authors declare that the research was conducted in the absence of any commercial or financial relationships that could be construed as a potential conflict of interest.

## Publisher’s note

All claims expressed in this article are solely those of the authors and do not necessarily represent those of their affiliated organizations, or those of the publisher, the editors and the reviewers. Any product that may be evaluated in this article, or claim that may be made by its manufacturer, is not guaranteed or endorsed by the publisher.
