# Supplementary material for: Qualitative analysis of front-of package labeling policy interactions between stakeholders and Health Canada
Source: Front Public Health. 2023 Apr 6;11:982908. doi: 10.3389/fpubh.2023.982908 (PMC10115994; doi:10.3389/fpubh.2023.982908)
Supplement: Supplementary file 1 [file Data_Sheet_1.PDF]

Supplementary Table 1. Description of the Thematic Framework Topic

Topic theme	Codes included in theme* (*codes in **bold**)
**Industry role as a stakeholder**	Industry stakeholders' responses including their willingness to employ **voluntary measures**, previous **achievements**, and the perceived unique **benefits** they provide.
**Industry engagement in policy development**	Industry expectations to be involved in the **development of the policy**, have regular **communication**, and be involved in the **decision making**.
**Concerns about the policy**	Little discussion of the **potential benefits** of the policy, and more focus on **concerns** about **unintended consequences** and the **economic impact** and **pressure on the food industry**.
**Consumers response to the policy**	**Consumer education** was described as a priority by all stakeholders, but particularly industry stakeholders who also had concerns that consumers would be **confused** by the current policy.
**Industry perception of a weak evidence base for the policy**	Industry stakeholders questioned the **evidence base** for the FOPL policy and argued for a need for **further research**. There was limited resistance to these claims by non-industry stakeholders.
**Global policy comparisons**	Many stakeholders commented on the potential for Canadian agri-food industry to take a **leading role** or **remain in tune** with partners by adopting industry-backed transnational FOPL policies rather than **lagging behind** if the proposed policy was implemented.
**Practicalities of policy implementation**	Discussion of the technical aspects of FOPL implementation focused on **packaging restrictions**, **pre-set threshold, exemptions**, and **colour coding**.
**Front-of Package symbol**	There was significant discussion on whether the HC proposed **high-in** symbol would be the most appropriate compared to other suggested by industry such as '**Facts-UpFront'** and '**refer to the Nutritional Facts Table'**.
